# Nitrogen stable isotope analysis of sulfonamides by derivatization-gas chromatography-isotope ratio mass spectrometry

**DOI:** 10.1007/s00216-024-05361-2

**Published:** 2024-06-07

**Authors:** Qingyuan Dou, Aoife Canavan, Yuhao Fu, Leilei Xiang, Yu Wang, Xi Wang, Xin Jiang, Christopher Dirr, Fang Wang, Martin Elsner

**Affiliations:** 1 State Key Laboratory of Soil and Sustainable Agriculture, Institute of Soil Science, Chinese Academy of Sciences, No. 71 East Beijing Road, Nanjing, 210008 China; 2 Chair of Analytical Chemistry and Water Chemistry, School of Natural Sciences, Department of Chemistry, Technical University of Munich, Lichtenbergstr. 4, 85748 Garching, Germany; 3 University of Chinese Academy of Science, Beijing, 100049 China

**Keywords:** Derivatization-gas chromatography-isotope ratio mass spectrometry, Nitrogen stable isotope analysis, Sulfonamides, Antibiotics, (Trimethylsilyl)diazomethane

## Abstract

The continuous introduction of micropollutants into the environment through livestock farming, agricultural practices, and wastewater treatment is a major concern. Among these pollutants are synthetic sulfonamide antibiotics such as sulfamethoxazole, which are not always fully degraded and pose a risk of fostering antimicrobial resistance. It is challenging to assess the degradation of sulfonamides with conventional concentration measurements. This study introduces compound-specific isotope analysis of nitrogen isotope ratios at natural abundances by derivatization-gas chromatography hyphenated with isotope ratio mass spectrometry (derivatization-GC-IRMS) as a new and more precise method for tracing the origin and degradation of sulfonamides. Here, sulfamethoxazole was used as a model compound to develop and optimize the derivatization conditions using (trimethylsilyl)diazomethane as a derivatization reagent. With the optimized conditions, accurate and reproducible δ^15^N analysis of sulfamethoxazole by derivatization-GC-IRMS was achieved in two different laboratories with a limit for precise isotope analysis of 3 nmol N on column, corresponding to 0.253 µg non-derivatized SMX. Application of the method to four further sulfonamides, sulfadiazine, sulfadimethoxine, sulfadimidine, and sulfathiazole, shows the versatility of the developed method. Its benefit was demonstrated in a first application, highlighting the possibility of distinguishing sulfamethoxazole from different suppliers and pharmaceutical products.

**Graphical Abstract:**

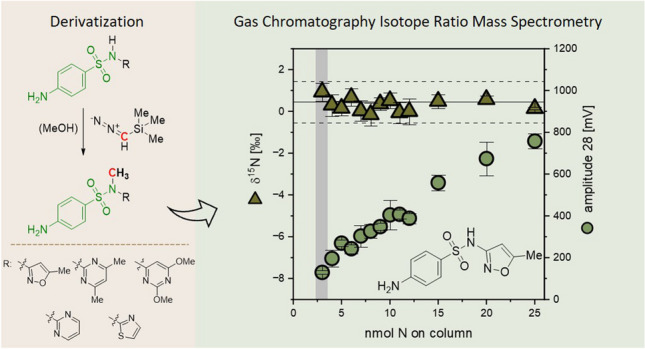

**Supplementary Information:**

The online version contains supplementary material available at 10.1007/s00216-024-05361-2.

## Introduction

The continuous introduction of antibiotics into the environment by livestock farming, agriculture, and wastewater discharge raises severe environmental and public health concern [1–3]. Antibiotics, as typical emerging contaminants, are often incompletely degraded, which can be harmful to living organisms [4]. The long-term accumulation of antibiotic residues accelerates the propagation and transfer of antibiotic-resistant genes and antibiotic-resistant bacteria [5, 6]. Sulfamethoxazole (SMX), a prevalent veterinary antibiotic employed for disease treatment and animal growth promotion [7], is found in an average concentration of 76 ng L^−1^ in investigated European rivers [8] and is frequently detected in feces [9] and wastewater effluents [10]. SMX can experience an array of biotic and abiotic transformations in the natural environment, including photodegradation, microbial degradation, adsorption/desorption, surface runoff conveyance, leaching, and plant uptake [11–15]. Understanding the transformation and degradation pathways of SMX is crucial for tracking its environmental impact. Currently, insights come from indirectly measuring residual concentrations and identifying transformation products. However, concentration measurements may be insufficient to understand substance degradation in complex environmental systems, as it is typically challenging to assess whether a decrease in concentrations stems from degradation or dilution [16], and to distinguish between different degradation mechanisms that would result in the same transformation products [17, 18]. Additionally, in questions of product authentication or source allocation, it is impossible to distinguish whether SMX originates from various sources based solely on concentration measurements. Therefore, there is great interest in developing new strategies and methodologies to enhance our understanding of the sources and transformation pathways of SMX in our environment. Compound-specific isotope analysis (CSIA) is a powerful tool for elucidating the origin of target analytes and characterizing degradation pathways even without knowledge about the metabolites involved [19–23].

This approach relies on the measurement of isotopic effects during (bio)chemical reactions in the form of changes in isotope ratios – isotope fractionation – that are observable for different elements in the molecular average. Previous studies on SMX have reported isotopic enrichment factors of carbon for photolysis at different wavelengths [24, 25], in both aerobic and anaerobic biodegradation by *Microbacterium* sp. strain BR1 [24] and *Desulfovibrio vulgaris* Hildenborough [26], respectively, and in chemical oxidation with ozone and chlorine dioxide [27]. However, relying on isotopic information from a single element alone has been shown to have limited conclusiveness. On the one hand, masking effects, including source mixing or rate-limiting steps within reaction cascades, lead to variations in isotope fractionation even if the same underlying degradation reaction prevails [28, 29]. On the other hand, molecules with a high number of atoms from the same element lead to the situation that many of them are not involved in the reaction (e.g., SMX has ten carbon atoms and only one is at a reactive site) [30] causing a “dilution” of position-specific isotope effects in the compound average. In these cases, observed isotope enrichment factors will not accurately reflect the characteristic isotope effects expected for an underlying reaction mechanism.

In such a situation, analyzing an additional element may help for the following reasons. On the one hand, masking will typically influence the isotope effects of both elements to the same extent. Therefore, changes in isotope ratios, when plotted for one element relative to another, will result in a characteristic dual element isotope slope that can allow for the distinction of different transformation pathways irrespective of the extent of masking. On the other hand, when analyzing a second element, information can frequently be tapped from a smaller number of atoms (e.g., SMX has only three nitrogen atoms), meaning that isotope effects are less “diluted.” Establishing a multi-element stable isotope analysis may, therefore, effectively circumvent the limitation of single-element isotopes for individual compounds.

SMX possesses three nitrogen atoms in the amino, sulfonamide, and isoxazole groups, which are relevant potential reaction sites, highlighting the interest in a method for nitrogen isotope analysis. The commonly employed technique for measuring polar and non-volatile compounds, including SMX, is liquid chromatography hyphenated to isotope ratio mass spectrometry (LC-IRMS) [24, 25, 27, 31]. However, LC-IRMS relies on a quantitative conversion of analyte molecules to the respective measurement gas in the liquid phase [32]. While this works well for CO_2_ in the case of carbon isotope analysis, no such method exists for the quantitative liquid-phase conversion of nitrogen-containing compounds to N_2_. The situation is different in the case of gas chromatography-isotope ratio mass spectrometry (GC-IRMS), where interfaces for carbon, nitrogen, hydrogen, and oxygen analysis are commercially available. Recently, Quang et al. [26] presented a GC-IRMS method for carbon and hydrogen isotope analysis of SMX. However, their gas chromatographic method reports the formation of three byproducts due to the thermal decomposition of SMX, illustrating the handicap that polar compounds like SMX are challenging to analyze via GC in undecomposed form. To overcome the limitations of LC-IRMS and to improve the compound stability in GC-IRMS, here we spearhead derivatization-gas chromatography-isotope ratio mass spectrometry (derivatization-GC-IRMS) as an alternative strategy. Successful implementations of nitrogen isotope analysis by derivatization-GC-IRMS have been shown for the analysis of several micropollutants and selected transformation products, including diclofenac [17], glyphosate [33], and desphenylchloridazon [34]. In this approach, derivatization is instrumental in reducing the polarity and increasing the thermal stability of a compound, making it GC-amenable. For isotope analysis, three aspects are particularly relevant in this context. (i) Quantitative conversion of the target compound is needed to avoid isotope effects during the reaction. (ii) As few atoms as possible should be introduced to minimize bias from external atoms. (iii) The derivatization procedure should be reproducible to keep the isotope effect of the externally introduced atoms as constant as possible [35]. This is why methylation – introducing only one further carbon atom – of the sulfonamide functionality is a promising strategy. Methylation of SMX using diazomethane has already been reported for residue analysis with GC–MS in eggs and animal tissues [36], but has never been explored for CSIA. Here, we, therefore, investigated the potential of derivatization-GC-IRMS using (trimethylsilyl)diazomethane (TMSD) as a less explosive derivatization reagent compared to diazomethane [37] for nitrogen isotope analysis of sulfonamides.

The overall goal was to develop and validate a derivatization-GC-IRMS method for different sulfonamide antibiotics. The specific objectives of our work were (i) to investigate the suitability of TMSD as a derivatization reagent of SMX for GC analysis; (ii) to assess the method detection limits, accuracy, and reproducibility of nitrogen isotope analysis and to explore limitations for carbon isotope analysis; (iii) to expand the developed derivatization-GC-IRMS method to additional sulfonamide antibiotic structures, namely, sulfadiazine, sulfadimethoxine, sulfadimidine, and sulfathiazole; and (iv) to finally apply the derivatization procedure for authentication purposes of SMX produced by different suppliers. To the best of our knowledge, this work demonstrates the first derivatization-GC-IRMS method of sulfonamides for nitrogen isotope analysis.

## Experimental section

### Chemicals and standard solutions

A detailed list of the chemicals and materials used, along with the purity of the gases, is provided in the supporting information (see Electronic Supplementary Material S1). Stock solutions of the respective target analyte were prepared and stored at − 20 °C until further usage.

### Derivatization procedure

**Scheme 1:**
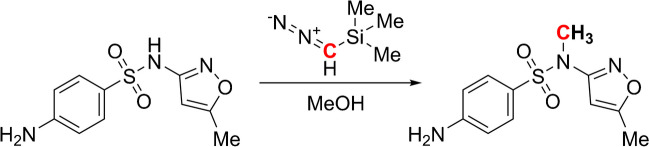
Derivatization reaction of SMX with TMSD and methanol

Following a procedure of Melsbach et al. [34], the methylation of sulfonamides using TMSD was accomplished at the N1 position (Scheme 1). The optimized derivatization procedures for SMX from two different laboratories are described here: *At CAS:* To 4-mL brown glass vials, 1 mL of the respective concentration (1.58 to 7.91 mM) was added. After adding TMSD in ether (1.5 M, 76 eq., corresponding to 120 to 400 µL), the vial was tightly closed and placed in an oven protected from light at 60 °C for 2 h. After the reaction, the solvent was evaporated to dryness under a gentle stream of nitrogen (r.t., ND200, Hangzhou Ruicheng Instrument Co., Ltd., China). The residue was reconstituted with 1 mL of acetone and vortexed at 3000 rpm for 1 min. All samples were passed through an organic filter (Nylon, 13 mm × 0.22 μm, PALL, USA) to 2 mL brown glass vials and stored at –20 °C for further analysis. *At TUM:* To 20-mL headspace vials, 1 mL of the respective SMX concentration (0.33 to 2.78 mM) was added. After adding TMSD in hexanes (2 M, ≥ 160 eq., 160 µL), the vials were tightly crimped and placed in a water bath at 50 °C for 1 h. Next, the reaction mixture was cooled to r.t., and the solvent was evaporated to complete dryness under a gentle stream of nitrogen (0.5 mL min^−1^, r.t., TurboVapLV, Biotage, Sweden). The residue was reconstituted with 1 mL of methanol and transferred to a 2-mL brown glass vial for isotope analysis. This method was also used for the derivatization of sulfadiazine, sulfadimethoxine, sulfadimidine, and sulfathiazole. For the development of the optimal derivatization conditions, 1 mL of SMX in MeOH (100 mg L^−1^) was derivatized screening different reaction temperatures (r.t., 50 °C and 60 °C), time spans (1 h, 2 h, and 3 h), and equivalents of TMSD (100, 150, 200, and 240) in triplicates. To this end, the yield of the derivatized product was quantified by GC–MS using an authentic standard of N1-methyl-sulfamethoxazole (SMX-Me).

### Analytical methods

#### EA-IRMS

*At CAS:* The nitrogen isotope values of SMX standards from different manufacturers were determined by an elemental analyzer-isotope-ratio mass spectrometer (EA-IRMS) system consisting of a Flash EA 1112 elemental analyzer and a Delta V Plus IRMS interfaced via a ConFlo IV (all, Thermo Fisher Scientific, USA). The isotope composition was determined through normalization against the reference material IAEA600 (Caffeine). *At TUM:* The determination of the nitrogen isotope compositions of our in-house standards sulfadiazine, sulfadimethoxine, sulfadimidine, sulfathiazole, and SMX, and commercially purchased SMX-Me was performed on an EA-IRMS system consisting of a EuroEA (Euro Vector, Italy), which was hyphenated to a Finnigan MAT 253 IRMS via a Finnigan ConFlo III interface (both, Thermo Fisher Scientific, Germany). The isotope ratios of samples and the working standard acetanilide were calibrated against the reference materials USGS40 (L-glutamic acid) and USGS41 (L-glutamic acid). The carbon  $\left({\delta }^{13}\text{C}\right)$- and nitrogen  $\left({\delta }^{15}\text{N}\right)$-isotope values (‰) are reported relative to PeeDee Belemnite (V-PDB) and air, respectively, according to the following Eqs. 1 and 2:

1 $$\delta {}_{ }{}^{13}\text{C}=\frac{{}_{ }{}^{13}\text{C}/{{}_{ }{}^{12}\text{C}}_{\text{sample}}-{}_{ }{}^{13}\text{C}/{{}_{ }{}^{12}\text{C}}_{\text{reference}}}{{}_{ }{}^{13}\text{C}/{{}_{ }{}^{12}\text{C}}_{\text{reference}}}$$

2 $$\updelta {}_{ }{}^{15}\text{N}=\frac{{}_{ }{}^{15}\text{N}/{{}_{ }{}^{14}\text{N}}_{\text{sample}}-{}_{ }{}^{15}\text{N}/{{}_{ }{}^{14}\text{N}}_{\text{reference}}}{{}_{ }{}^{15}\text{N}/{{}_{ }{}^{14}\text{N}}_{\text{reference}}}$$

#### GC-IRMS

The GC-IRMS procedures of both laboratories are briefly described here; more details can be found in the supporting information (see Electronic Supplementary Material S2). *At CAS:* The nitrogen isotope ratios of derivatized SMX were determined on a GC-IRMS system consisting of a TRACE 1310 gas chromatograph, which was connected through a GC IsoLink II and a ConFlo IV interface to a Finnegan MAT 253 Plus isotope ratio mass spectrometer (all, Thermo Fisher Scientific, Germany). For the analysis, 4 µL of the sample was injected by a TriplusRSH autosampler (Thermo Fisher Scientific, Germany) into a split/splitless injector. The injector was equipped with a splitless liner and maintained at a temperature of 250 °C. The target analyte was separated on an HP-5MS column (30 m length, 0.25 mm ID, 0.25 μm film thickness, Agilent Technologies, USA) at a constant helium flow rate of 1.0 mL min^−1^. The GC temperature procedure was as follows: The initial temperature of 80 °C was held for 2 min, increased to 180 °C at 30 °C min^−1^ and held for 2 min, increased to 280 °C at 10 °C min^−1^ and held for 5 min. For combustion of the target analyte, commercial combustion reactors (NiO tube CuO − NiO reactor, Thermo Fisher Scientific, Germany) were used at a temperature of 1020 °C. *At TUM:* The $\left({\delta }^{15}\text{N}\right)$ isotope ratio analysis was conducted on a GC-IRMS system consisting of a Trace 1310 gas chromatograph coupled to a Finnigan MAT 253 isotope ratio mass spectrometer via a GC IsoLink II and a ConFlo IV interface (all, Thermo Fisher Scientific, Germany). For the analysis, samples of 3 µL were injected by a TriPlusRSH (Thermo Fisher Scientific, Germany) into a split/splitless injector equipped with a splitless liner (4 mm ID × 78.5 mm length, 800 µL, ultra inert, double taper, without glass wool, Agilent Technologies, USA) maintained at a temperature of 250 °C. Initially, the injector was operated in splitless mode with a surge pressure of 250 kPa for 1 min. Subsequently, it was switched to split mode, maintaining a split flow rate of 20 mL min^−1^. The target analytes were separated at a constant helium flow rate of 1.4 mL min^−1^ on an Agilent J&W DB-5MS UI column (30 m length × 0.25 mm ID × 1.0 μm film thickness, Agilent Technologies, USA), which was enclosed by a pre-and post-column (each 1 m deactivated fused silica guard column, 0.25 mm ID, Agilent, USA). The GC oven program started at 120 °C (held for 1 min), followed by a first ramp to 250 °C with a rate of 22 °C min^−1^ and a second ramp to 325 °C with a rate of 40 °C min^−1^, where the final temperature was held for 11 min. Upon elution from the column, the target analytes underwent combustion and subsequent reduction at a temperature of 1000 °C using commercial combustion reactors (NiO tube CuO − NiO reactor, 2 mm, Thermo Fisher, Germany). The respective limits of precise isotope analysis and the method’s trueness were determined using varying concentrations of the respective sulfonamides for derivatization (19 to 95 nmol N on column *at CAS* and 2 to 25 nmol N on column *at TUM* assuming quantitative conversion). The software Isodat (Thermo Fisher Scientific, Germany) automatically performed peak detection and integration using the individual background algorithm for baseline correction. The method quantification limits were determined with the moving mean procedure according to Jochmann et al. [38].

#### GC–MS

GC–MS measurements were performed on an Agilent 7890A GC system equipped with a 5975C Triple-Axis Detector MS (all, Agilent Technologies, USA) *at TUM*. For the analysis, samples of 2 µL were injected by a Combi PAL-XT autosampler (CTC Analytics AG, Switzerland) into a split/splitless injector equipped with a splitless liner with glass wool (4 mm ID × 80 mm length, Trajan, Germany) maintained at a temperature of 250 °C with a pressure of 0.9 bar, a total flow of 19.4 mL min^−1^ and a septum purge flow of 3 mL min^−1^. The target analytes were separated on an Agilent J&W DB-5MS column (30 m length × 0.25 mm ID × 1.00 µm film thickness, Agilent Technologies, USA) at a constant helium flow rate of 1.4 mL min^−1^. The column was enclosed by a pre-and post-column (each 1 m deactivated fused silica guard column, 0.25 mm ID, Agilent, USA). The end of the post-column was kept at a constant temperature of 250 °C during the whole measurement. A GC oven program of 60 °C (hold: 1 min) and a ramp of 10 °C min^−1^ to 300 °C (hold: 13 min) was used to determine the yield of SMX-Me during method optimization. Ionization was accomplished with a high-efficiency source (HES) at 70 eV. The quadrupole and ion source temperatures were set to 150 °C and 230 °C, respectively. From each sample, a full scan spectrum over a mass range of 40 to 1000 and triplicates in single ion mode (SIM) for quantification were recorded using caffeine (10 mg L^−1^) as an internal standard. As quantifying ions, m/z 92 and 194 were used for both SMX-Me and its byproduct 4-amino-*N*-methylbenzenesulfonamide and caffeine, respectively. Data were analyzed by MSD ChemStation E.02.02.1431 (Agilent Technologies, USA). The measured compound mass spectra were compared with a database (NIST MS Search 2.0, USA).

### Extraction of sulfamethoxazole from pharmaceuticals

Solid-phase extraction (SPE) was used to extract and concentrate SMX from veterinary pills *at CAS*. Before the SPE procedure, to a centrifuge tube (polypropylene, 50 mL), the ground pharmaceutical (25 mg) and Na_2_EDTA-McIlvaine buffer (20 mL, 0.1 M, pH 4) were added. Next, the suspension was homogenized by vortexing for 1 min, followed by ultrasonication for 30 min at 20 °C. After homogenization, the samples were centrifuged at 4000 rpm for 10 min. The supernatant containing the desired SMX was transferred into a second centrifuge tube. The residual was extracted for a second time according to the procedure described above. The polar enhanced polymer (PEP) cartridges (6 mL, 200 mg, Agela Technologies, China) were conditioned with methanol (10 mL) followed by ultrapure water (10 mL). Next, the combined supernatant from both extractions was passed through the pre-conditioned cartridges at a flow rate of 5–10 mL min^−1^. After loading the samples on the cartridges and washing them with ultrapure water (5 mL), the cartridge was dried under a gentle nitrogen stream for 10 min. The sample was eluted with methanol (3 × 2 mL). The combined eluates were concentrated under a gentle stream of nitrogen (r.t., ND200, Hangzhou Ruicheng Instrument Co., Ltd, China). The respective recoveries were determined by high-performance liquid chromatography (see Electronic Supplementary Material S3).

## Results and discussion

### Derivatization of SMX and GC–MS analysis

**Fig. 1:**
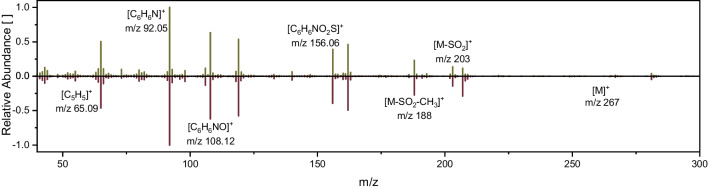
Comparison of the mass spectra of derivatized SMX (green, top) and the authentic standard SMX-Me (burgundy, bottom), including annotations of assigned mass fragments (proposed structures see Electronic Supplementary Material Fig. S1)

The derivatization of SMX with TMSD resulted in the methylated derivative of SMX, N1-methyl-sulfamethoxazole (SMX-Me), as the major product of the reaction (Scheme 1), which was verified by GC–MS analysis. The mass spectra of derivatized SMX and the authentic standard of SMX-Me show the same mass fragmentation pattern (Fig. 1). They agree with the mass spectrum of SMX-Me synthesized from SMX and diazomethane from Takatsuki et al. [36]. In these mass spectra, the molecular ion [M]^+^ is present at a low abundance. In contrast, the observable major fragments are [M-SO_2_-CH_3_]^+^ at m/z 188, [C_6_H_6_NO_2_S]^+^ at m/z 156, [C_6_H_6_NO]^+^ at m/z 108, [C_6_H_6_N]^+^ at m/z 92, and [C_5_H_5_]^+^ at m/z 65. Here, the fragment [M-SO_2_]^+^ at m/z 203 is the most characteristic, since it contains the additional methyl group. For method development and optimization of the derivatization procedure, SMX-Me was injected into our GC–MS system at different concentrations, showing a broad linear range (see Electronic Supplementary Material Fig. S2a). The authentic standard and derivatized SMX show the formation of 4-amino-N-methylbenzenesulfonamide as a byproduct, corresponding to a byproduct-to-SMX ratio of approximately 1/30. The byproduct’s response linearly increased with increasing amount of injected SMX-Me (see Electronic Supplementary Material Fig. S2b), indicating its formation during injection or on the column. Comparing this derivatization approach to the one without derivatization by Quang et al. [26], the number of byproducts was reduced to one, and the lowest detected concentration was reduced from 1.3 to 0.04 g L^−1^. For method optimization, different reaction temperatures (r.t., 50 °C and 60 °C) and reaction times (1 h, 2 h, and 3 h) were investigated at a constant molar ratio of TMSD-to-analyte (240) (see Electronic Supplementary Material Fig. S3a). The influence of temperature and time exhibited minimal variations at higher temperatures, underscoring the method’s robustness. However, at room temperature, a significant decrease in recovery was observed with prolonged reaction times. Additionally, various molar ratios of TMSD-to-analyte (100, 150, 200, and 240) were compared using the optimized conditions, revealing no significant differences in recoveries (see Electronic Supplementary Material Fig. S3b). A reaction temperature of 50 °C and a reaction time of 1 h showed a yield of SMX-Me of up to 87% and was selected for further experiments *at TUM*.

### GC-IRMS method validation and limit of precise isotope analysis of SMX

**Fig. 2:**
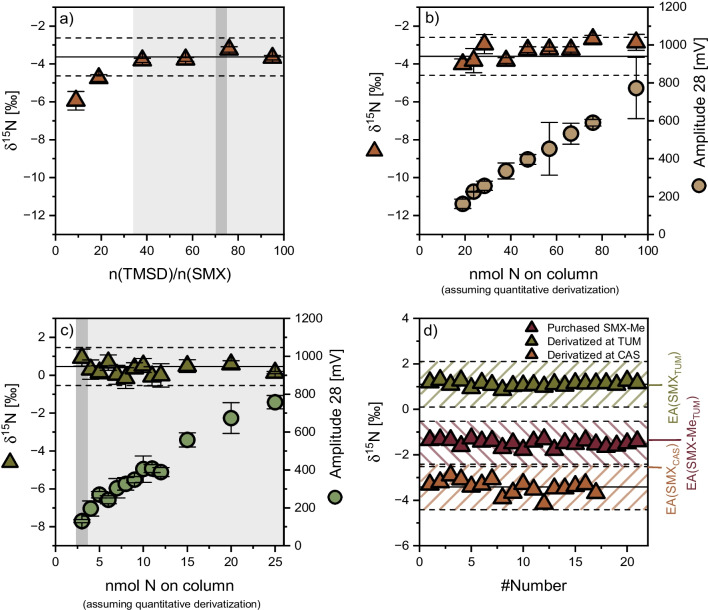
Nitrogen isotope analysis of derivatized SMX; **a** ${\delta }^{15}\text{N}$ values showing dependence on the molar ratio of TMSD-to-analyte used in the derivatization procedure *at CAS* (the light grey area indicates the range of stable isotope measurement; dark grey bar represents the excess of TMSD used for part the method validation); **b** + **c** ${\delta }^{15}\text{N}$ and amplitudes showing dependence on the nmol N on column assuming quantitative conversion during the derivatization process **b** *at CAS* (using 76 eq. of TMSD); **c** *at TUM* (using ≥ 160 eq. of TMSD); linear ranges of the method are displayed in light grey, with the dark grey bar showing the method quantification limit; and **d** reproducibility of ${\delta }^{15}\text{N}$ values from derivatized SMX and purchased SMX-Me measured with GC-IRMS, *at CAS* multiple injections over 3 months and *at TUM* one measurement sequence, annotating the EA-IRMS values on the right in the respective color; note: in all graphs, the black solid lines show the calculated moving means (**c** + **b**) or mean value (**a** + **d**) encompassed by a ± 1‰ interval (black dashed lines)

**Fig. 3:**
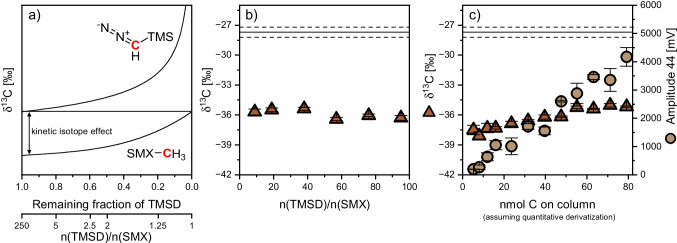
**a** Expected trends in carbon isotope ratios of the methyl equivalent in TMSD (upper graph) and the derivative (lower graph) illustrating that isotope values of external carbon vary depending on derivatization conditions, TMS = trimethylsilyl. **b** + **c** Comparison with the variability of ${\delta }^{13}\text{C}$ in dependence on TMSD excess and the substance amount on column

The method for $\delta^{15}\text{N}$ isotope analysis was validated, and the method quantification limit was determined in the two laboratories. *At CAS*, the effect of a different excess of TMSD on the ${\delta }^{15}\text{N}$ value was investigated by varying the molar ratio of TMSD-to-SMX from 9 to 95 (Fig. 2a). Here, a plateau of the $\delta^{15}\text{N}$ value was reached at an excess of TMSD greater than 38. In contrast, a molar ratio of TMSD-to-analyte lower than 38 resulted in incomplete conversion of SMX to SMX-Me and, therefore, bias in $\delta^{15}\text{N}$ values. In the next step, different concentrations ranging from 19 to 95 nmol N on column of SMX were derivatized to explore the method quantification limit (Fig. 2b). As expected, the limit of precise nitrogen isotope analysis of SMX was amplitude-dependent, and a threshold of 100 mV for amplitude 28 was determined (see Electronic Supplementary Material Fig. S4). *At CAS*, this corresponded to a limit of 19 nmol N on column or 1.60 µg non-derivatized SMX. *At TUM*, following the approach of Melsbach et al. [34], the method validation was performed with an excess of 160 of TMSD using a concentration range from 3 to 25 nmol N on column (Fig. 2c). Here, the limit for precise isotope analysis was more than six times lower, namely, 3 nmol N, corresponding to 0.253 µg non-derivatized SMX. The discrepancy in the determined limits between both laboratories can be attributed to the peak width, which was half as wide *at TUM* compared to *CAS* (see Electronic Supplementary Material Fig. S5). To ensure the trueness of isotope measurements, best practice in standardization for CSIA was adhered to. Standards need to correct for (i) effects in ionization and IRMS instrumentation, (ii) oxidation efficiency, and (iii) complete conversion during the derivatization reaction. In contrast to common practice in concentration analysis by GC–MS, isotopically labeled internal standards cannot be used in GC-IRMS measurements to correct for (i) because this would change the isotope results. We, therefore, relied on external standardization. To control for (ii), caffeine was used as standard analyzed alongside our target analyte in each chromatographic run. The influence of (iii) was carefully controlled by optimizing the derivatization procedure beforehand (see above). The ${\delta }^{15}\text{N}$ values measured on GC-IRMS in this way were compared to the respective ${\delta }^{15}\text{N}$ EA-IRMS values (Fig. 2d). If deviations in isotope values are observed, they often depend on the conditions in the respective combustion reactor. Such deviations are not of concern as long as they are reproducible and corrections can be made to adjust them [39]. *At TUM*, the ${\delta }^{15}\text{N}$ value measured by derivatization-GC-IRMS showed no deviation from the EA-IRMS for both the derivatized SMX ($\delta^{15}{\mathrm N}_{\mathrm{GC}-\mathrm{IRMS}}=\;+1.1\;\pm\;0.1\backslash\mathrm{permille}\;\mathrm{vs}.\;\delta^{15}{\mathrm N}_{\mathrm{EA}-\mathrm{IRMS}}=\;+1.0\;\pm\;0.1\backslash\mathrm{permille}$) and the purchased SMX-Me ($\delta^{15}{\mathrm N}_{\mathrm{GC}-\mathrm{IRMS}}=\;-1.5\;\pm\;0.2\backslash\mathrm{permille}\;\mathrm{vs}.\;\delta^{15}{\mathrm N}_{\mathrm{EA}-\mathrm{IRMS}}=\;-1.4\;\pm\;0.1\backslash\mathrm{permille}$) (Fig. 3d), indicating no isotope fractionation during derivatization and conversion in the combustion reactor of the GC-IRMS. *At CAS*, a reproducible deviation of 0.7‰ ($\delta^{15}{\mathrm N}_{\mathrm{GC}-\mathrm{IRMS}}=\;-3.2\;\pm\;0.1\backslash\mathrm{permille}\;\mathrm{vs}.\;\delta^{15}{\mathrm N}_{\mathrm{EA}-\mathrm{IRMS}}=\;-2.5\;\pm\;0.0\backslash\mathrm{permille}$) was observed, which is likely caused by the respective combustion reactor and can be easily corrected for.

It was also tested whether the developed derivatization method can result in reliable carbon isotope analysis. Unlike for ${\delta }^{15}\text{N}$ isotope analysis, potential bias can be expected, since the methyl group introduces an additional atom of the element of interest (i.e., carbon). Further, since TMSD is added in great excess, it is impossible to achieve quantitative conversion of the derivatization agent by design – rather, only a small fraction will make its way into the methyl group in the derivative (Fig. 3 and see Electronic Supplementary Material Fig. S6). Hence, the question arises whether the derivatization procedure can be designed in a reproducible way to keep the isotope effect of this externally introduced carbon as constant as possible. Problems arise by the facts (i) that TMSD is highly reactive and has a limited shelf-life so that its isotopic composition may change over time and (ii) that it is difficult to keep the TMSD excess constant when samples contain a background matrix that is also methylated and uses up the derivatization agent. Indeed, Fig. 3 shows that, unlike for nitrogen, the ${\delta }^{13}\text{C}$ values significantly differed from their EA value, showed no stable linear range, and changed by as much as 3‰ depending on derivatization conditions. Considering that the position-specific changes in the isotope ratio of the methyl group are diluted by the constant isotope values of the other ten carbon atoms in SMX, this group’s values must have changed by even more than 30‰. While these results suggest that our derivatization approach with TMSD is not particularly reliable for carbon isotope analysis at natural isotopic abundance, they illustrate its excellent precision when ^13^C labeled SMX is addressed, such as in stable isotope labeling experiments.

### Application of the developed method to further sulfonamides

**Fig. 4:**
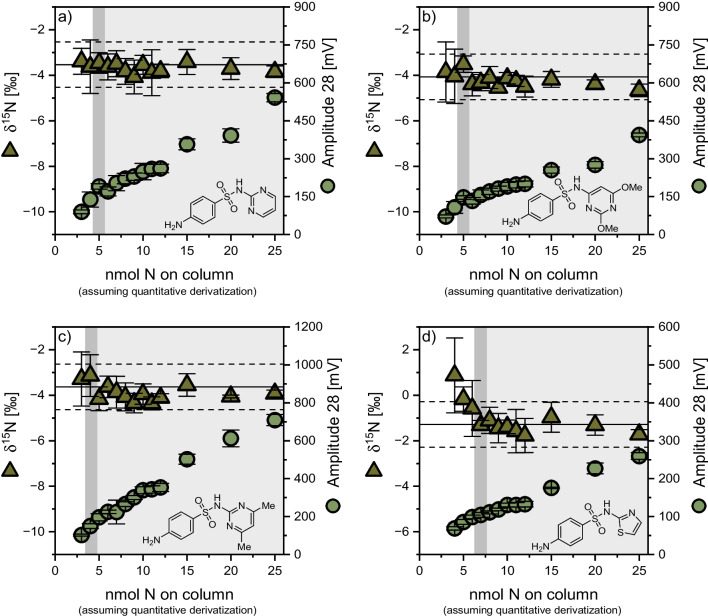
Amount-dependency of accurate nitrogen isotope ratio determination of the in-house standards: **a** sulfadiazine, **b** sulfadimethoxine, **c** sulfadimidine, and **d** sulfathiazole *at TUM*; note: linear ranges of the method are displayed in light grey, with the dark grey bar showing the method quantification limit; calculated moving means are represented by black solid lines encompassed by a ± 1‰ interval (black dashed lines); the chemical structures show the compound before derivatization

The developed method was further applied to four additional sulfonamide antimicrobials, namely sulfadiazine, sulfadimethoxine, sulfadimidine, and sulfathiazole, and the respective method quantification limits were determined (Fig. 4). The obtained method quantification limits are higher than those found for SMX. For sulfadiazine and sulfadimethoxine, a limit of 5 nmol N on column was found, whereas the limit for sulfadimidine was 4 nmol N on column. Sulfathiazole showed the highest limit of quantification with 6 nmol N on column assuming quantitative conversion. As for SMX in the *CAS* laboratory, a constant deviation from their respective EA-values was found for these four compounds, ranging from 0.5 to 1.9‰ (see Electronic Supplementary Material Table S3), which can be corrected for, because unlike for carbon in Fig. 3, the offset is amount-independent. A plausible reason for this would be incomplete combustion, as stated by Melsbach et al. [34]. The four further examples show the versatility of the developed derivatization-GC-IRMS method, which likely stands representative of other sulfonamides.

### Source fingerprinting and authentication of SMX

Stable isotope analysis has already proven valuable for fingerprinting to authenticate products and trace origins for various pharmaceuticals, including aspirin, diclofenac, ibuprofen, and naproxen [17, 40, 41]. In a specific case, it even provided evidence for the location of an illegal production site [42]. Therefore, isotope analysis is crucial for differentiating SMX sources in the environment, such as animal manure and wastewater effluents. To the best of our knowledge, for SMX, only a method for carbon isotope analysis has been developed, which revealed very small variations in the carbon isotopic signature of less than 1‰ when investigating six different pharmaceutical products [31]. Since nitrogen isotope analysis has been shown to be a very powerful tool for source identification of micropollutants in the environment [43], we undertook a survey to assess the typical nitrogen isotope ratio variations in SMX produced from different suppliers (Table 1, see Electronic Supplementary Material Table S4). The ${\delta }^{15}\text{N}$ values show a significant variability ranging from + 1.1 to − 7.0‰, highlighting the potential of using the ${\delta }^{15}\text{N}$ value of SMX as an addition to carbon isotope values for fingerprinting to trace the parent compound SMX. Furthermore, our approach facilitates isotope analysis of SMX in matrices like surface waters without tedious sample preparation (see Electronic Supplementary Material Table S5), underlining its applicability for real-world samples.

**Table 1:** Nitrogen isotope values of SMX from five different suppliers (A–E) and three pharmaceutical products (F–H) by EA-IRMS and derivatization-GC-IRMS

Entry	Laboratory	${\delta }^{15}{\text{N}}_{\text{EA}-\text{IRMS}}$ [‰]	${\delta }^{15}{\text{N}}_{\text{GC}-\text{IRMS}}$ [‰]^*a*^
A	CAS	− 2.2 ± 0.1	− 2.2 ± 0.1
B	CAS	− 2.5 ± 0.0	− 2.5 ± 0.1
C	CAS	− 4.5 ± 0.1	− 4.7 ± 0.3
D	CAS	− 3.5 ± 0.0	− 3.5 ± 0.2
E	TUM	+ 1.0 ± 0.1	+ 1.1 ± 0.1
F	CAS	n.a.^*b*^	− 7.0 ± 0.4
G	CAS	n.a	− 6.5 ± 0.2
H	CAS	n.a	− 6.6 ± 0.5

^a^Corrected values for measurements *at CAS*

^b^*n.a.* not analyzed

## Conclusion

With derivatization-GC-IRMS, this study brings forward a new approach to accomplish reproducible, precise, and true compound-specific nitrogen stable isotope analysis of sulfamethoxazole (0.253 µg of SMX on-column for nitrogen isotope analysis). It, therefore, opens a new avenue for studying not only different origins but also transformation mechanisms in natural and engineered systems [23] and accesses a new dimension for isotopic fingerprinting of sulfonamides at low levels to trace sources and detect counterfeiting. In its ability to characterize transformation mechanisms, the approach will be particular attractive for the optimization of advanced oxidation processes in water treatment where sufficient sample amounts are easily obtained in laboratory experiments and test stands. To inform about transformation processes also in natural systems, the previously reported average concentration of SMX of 76 ng L^−1^ in surface water needs to be made accessible to CSIA. Here, the combination of our method with recent advances in appropriate sample enrichment strategies [44, 45] brings compound-specific ${\delta }^{15}\text{N}$ values of SMX within reach in environmental water samples.

## Supplementary Information

Below is the link to the electronic supplementary material.

Supplementary file1 (PDF 700 KB)
