# Supplementary material for: Nitrogen stable isotope analysis of sulfonamides by derivatization-gas chromatography-isotope ratio mass spectrometry
Source: Anal Bioanal Chem. 2024 Jun 7;416(19):4237–47. doi: 10.1007/s00216-024-05361-2 (PMC11525405; doi:10.1007/s00216-024-05361-2)
Supplement: Supplementary file 1 — Supplementary file1 (PDF 700 KB) [file 216_2024_5361_MOESM1_ESM.pdf]

Supporting Information for *Analytical and Bioanalytical Chemistry*

Nitrogen stable isotope analysis of sulfonamides by derivatization-gas chromatography isotope ratio mass spectrometry

Qingyuan Dou [1,3 #], Aoife Canavan [2 #], Yuhao Fu [1,3], Leilei Xiang [1,3], Yu Wang [1,3], Xi Wang [1,3], Xin Jiang [1,3], Christopher Dirr [2], Fang Wang [1,3*], Martin Elsner [2 *]

[1] State Key Laboratory of Soil and Sustainable Agriculture, Institute of Soil Science, Chinese Academy of Sciences, Nanjing 210008, China

[2] Chair of Analytical Chemistry and Water Chemistry, School of Natural Sciences, Department of Chemistry, Technical University of Munich, Garching 85748, Germany

[3] University of Chinese Academy of Science, Beijing 100049, China

# shared first authorship: these authors contributed equally to the manuscript

*Corresponding author: Fang Wang
Address: No. 71 East Beijing Road, Nanjing 210008, China
Phone: +86-025-86881350
E-mail: wangfang@issas.ac.cn; fangwang321@issas.ac.cn
ORCID: 0000-0001-9986-0948

*Corresponding author: Martin Elsner
Address: Lichtenbergstr. 4 85748 Garching b. München
Phone: +49 (89) 289 - 54500
E-mail: m.elsner@tum.de

Contents

S1 Chemicals and Gases 2
S2 Additional Information on GC-IRMS 3
S3 Validation of the Extraction Procedure 4
S4 Structure Assignment of GC-MS Fragments 4
S5 Development and Optimization of the Derivatization Procedure 5
S6 Further Data on Derivatization-GC-IRMS for Nitrogen Isotope Analysis 6
S7 Derivatization-GC-IRMS for Carbon Isotope Analysis 7
S8 EA-IRMS vs. Derivatization-GC-IRMS of Further Sulfonamides 7
S9 EA-IRMS vs. Derivatization-GC-IRMS of SMX from Different Suppliers 8
S10 Recoveries and derivatization-GC-IRMS Analysis from Surface Water 8

S1 Chemicals and Gases

All chemicals used in the respective laboratories are summarized in Tables S1 and S2. *At CAS:* The GC-MS and GC-IRMS used helium (5.0, Nanjing Wenda Special Gases Co., Ltd, China, Country) as carrier gas. The reference gas used for nitrogen isotope analysis was nitrogen (5.0, Nanjing Wenda Special Gases Co., Ltd, China, Country). Nitrogen (5.0, Nanjing Wenda Special Gases Co., Ltd, China, Country) was used for evaporation. The ultrapure water used was purified from deionized water using a MilliQ unit (18.2 MΩ cm, MilliPore, Germany). *At TUM:* The GC-MS and GC-IRMS used helium (5.0, Westfalen, Germany) as carrier gas. The reference gas used for nitrogen isotope analysis was nitrogen (6.0, Westfalen, Germany). Nitrogen of lower purity (5.0, Westfalen, Germany) was used for evaporation. Stock solutions of each sulfonamide were prepared in acetone or methanol at concentrations ranging from 2 g L^{-1} to 5 g L^{-1}. They were stored at −20 °C and diluted with methanol to receive the desired concentration before derivatization.

Table S1: Summary of chemicals used *at CAS*.

Chemical	CAS number	Lot number	Further Information	Supplier
Analytical Standards				
Sulfamethoxazole	723-46-6	D0037162	99.7%	Dr. Ehrenstorfer
Sulfamethoxazole	723-46-6	G991644	99.6%	Dr. Ehrenstorfer
Sulfamethoxazole	723-46-6	V23EN-NI	> 98%	Tokyo Chemical Industry Co., Ltd.
Sulfamethoxazole	723-46-6	L960V11	98%	J&K Scientific
Pharmaceutical product containing Sulfamethoxazole	-	-	tablet	Anhui Kangmu Pharmaceutical Co., Ltd.
Pharmaceutical product containing Sulfamethoxazole	-	-	tablet	Shangdong Zhengmu Biological Pharma-ceutical Co., Ltd.
Pharmaceutical product containing Sulfamethoxazole	-	-	tablet	Huazhong Pharmaceutical Co., Ltd.
Reagents				
Trimethylsilyldiazo-methane	18107-18-1	-	1.5 M in hexanes	Sigma Aldrich
Solvents				
Acetone	67-64-1	-	HPLC-grade	Merck
Methanol	67-56-1	-	HPLC-grade	Merck

Table S2: Summary of chemicals used *at TUM*.

Chemical	CAS number	Batch number	Further Information	Supplier
Analytical Standards				
Me-Sulfamethoxazole	51543-31-8	823002	> 95%	VladaChem Gmbh
Sulfadiazine	68-35-9	BCCB6548	VETRANAL®, analytical standard	Sigma Aldrich
Sulfadimethoxine	122-11-2	BCCK4645	VETRANAL®, analytical standard	Sigma Aldrich
Sulfadimidine	57-68-1	BCCH7734	VETRANAL®, analytical standard	Sigma Aldrich
Sulfamethoxazole	723-46-6	BCCH3594	analytical standard	Sigma Aldrich
Sulfathiazole	72-14-0	BCCG6472	VETRANAL®, analytical standard	Sigma Aldrich
Reagents				
Trimethylsilyldiazo-methane	18107-18-1	-	2 M in hexanes	Thermo Fisher Scientific
Solvents				
Acetone	67-64-1	-	suitable for HPLC, ≥ 99.9%	Sigma Aldrich
Methanol	67-56-1	-	for HPLC, gradient grade, ≥ 99.9%	Sigma Aldrich

S2 Additional Information on GC-IRMS

At CAS: Before the nitrogen stable isotope ratio was determined by IRMS, a liquid N_2 cold trap device was used to remove the CO_2 converted from combustion. Derivatization was conducted in triplicate to ensure reliability. Following the analysis of about 120 samples, the oxidation furnace was oxidized for 60 minutes, and then the oxygen background was reduced by backflushing for 60 minutes. Calibration of the instrument was further ensured by analyzing the standard sample with a known isotope ratio. The N_2 reference gas was calibrated to nitrogen (Purity >99.9%, Lot no. 160-402153389-1, Air Liquide, France). *At TUM:* Between the combustion interface and the IRMS, the capillary carrying the He stream was emplaced into a liquid N_2 trap to freeze out the CO_2 generated during analyte combustion. This precautionary measure ensured that the CO_2 did not enter the ion source, preventing any potential interference of CO^+ ions with the measurement of N_2^+ isotopologues. The captured CO_2 was released daily from the trap by removing the capillary out of the liquid N_2. The oxidation capacity of the reactor was ensured before each sequence (~100 measurements) by oxidation for 60 min, followed by a subsequent backflush period of 60 min to allow oxygen levels to decrease before analysis. Before starting the sequence, standards were injected until stable isotope values were observed. The derivatization procedures were performed twice. Each replicate was measured in triplicate and bracketed with triplicates of a respective standard in concentrations corresponding

to 15 nmol N on column. To ensure system stability and method accuracy, retention times and isotope values were constantly monitored, and samples were bracketed with in-house standards. The N_2 reference gas was calibrated to air by reference materials (IAEA600, USGS 62, USGS 63).

S3 Validation of the Extraction Procedure

Concentrations of SMX in extracts were measured by high-performance liquid chromatography (HPLC) (Agilent 1260, USA) equipped with a diode array detector (DAD) set to 270 nm. The analyte separation was achieved by a symmetry C18 Column (4.6 × 250 mm, 5 µm, Waters Corporation, USA), which was kept at a temperature of 35 °C, and the flow rate was maintained at 1 mL min^{-1}. The initial mobile phase consisted of 90% water containing 0.1% formic acid (A) and 10% methanol (B). The amount of solvent B increased to 90% within 8 min, returned to initial settings after 2 min, and maintained there for 2 min. The injected volume was 10 µL. The recovery of the procedure was tested for extraction of SMX from commercial pharmaceutical products and exceeded 80%.

S4 Structure Assignment of GC-MS Fragments

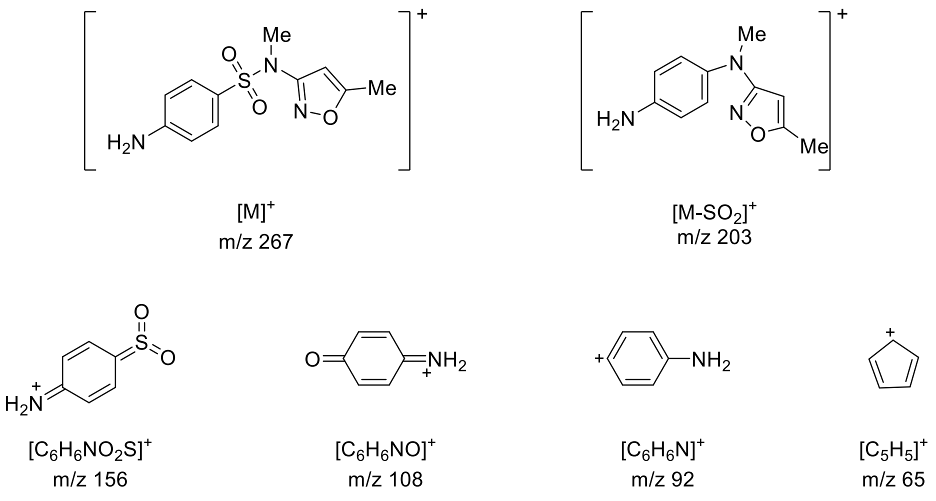

Figure S1: Proposed chemical structures of fragments found in the mass spectra of both the derivatized SMX and purchased SMX-Me.

S5 Development and Optimization of the Derivatization Procedure

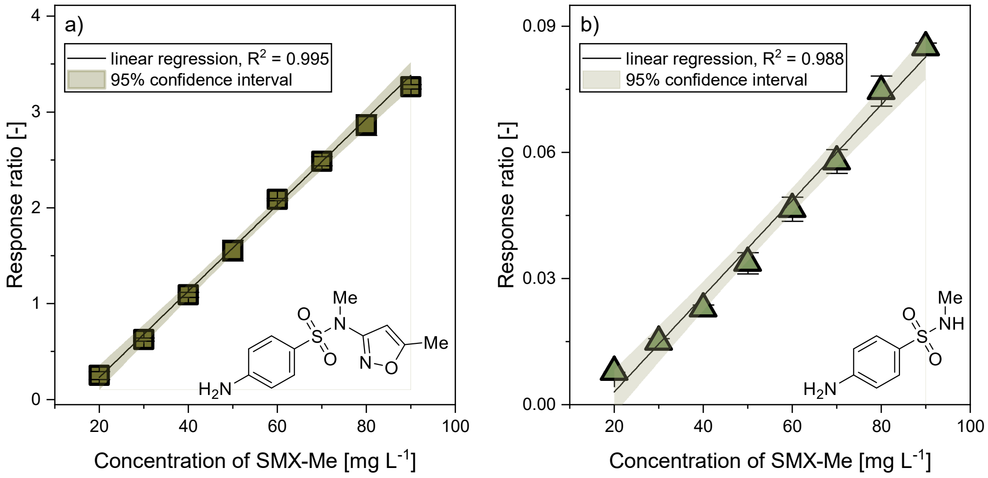

Figure S2: Calibration curves of a) commercially purchased SMX-Me and b) the by-product formed during GC-MS measurement using caffeine as an internal standard.

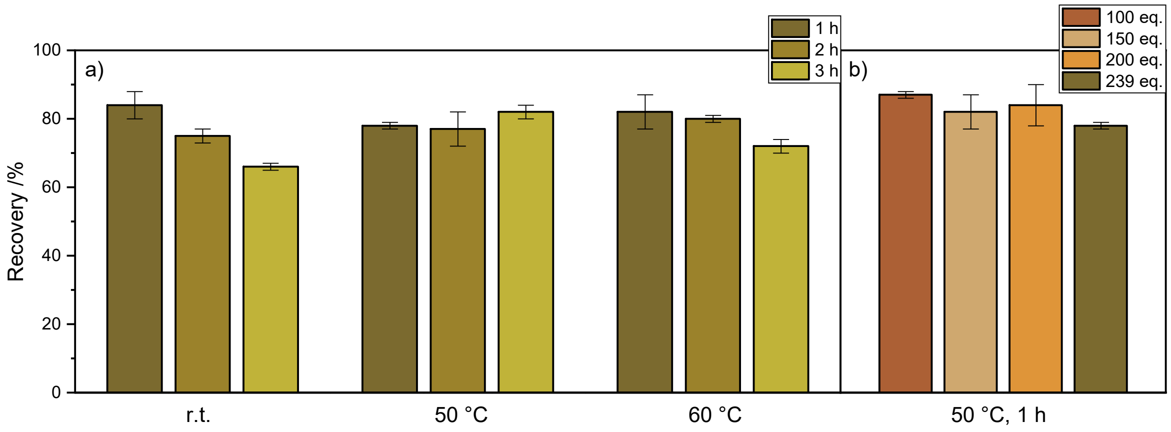

Figure S3: Recoveries of SMX-Me determined by GC-MS using caffeine as an internal standard *at TUM*, a) investigating different reaction temperatures and times; b) different molar ratios of TMSD-to-analyte.

Further Data on Derivatization-GC-IRMS for Nitrogen Isotope Analysis

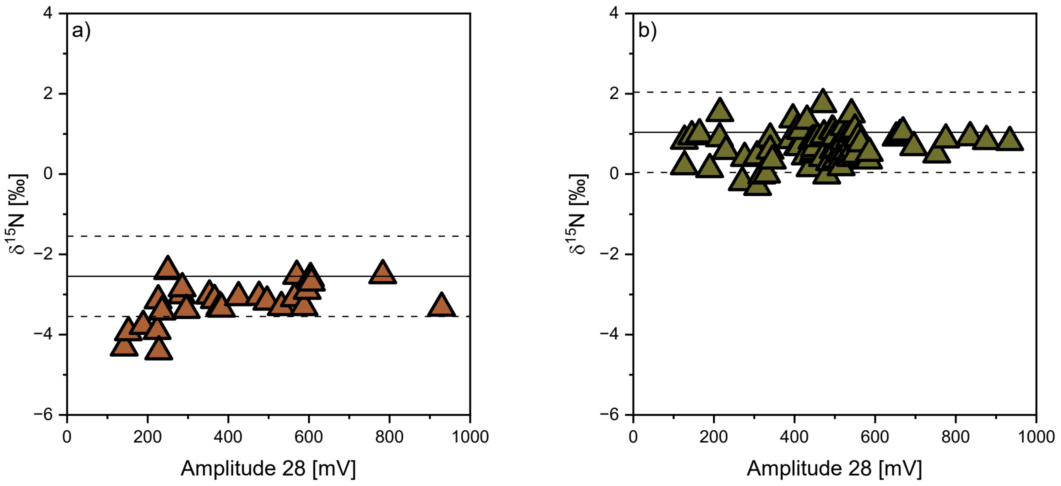

Figure S4: $\delta^{15}N$ showing dependence on the amplitudes a) *at CAS* and b) *at TUM*; note: in all graphs, the black lines show the EA-IRMS value of non-derivatized SMX and its tolerated standard deviation of ±1 ‰ (black dashed lines).

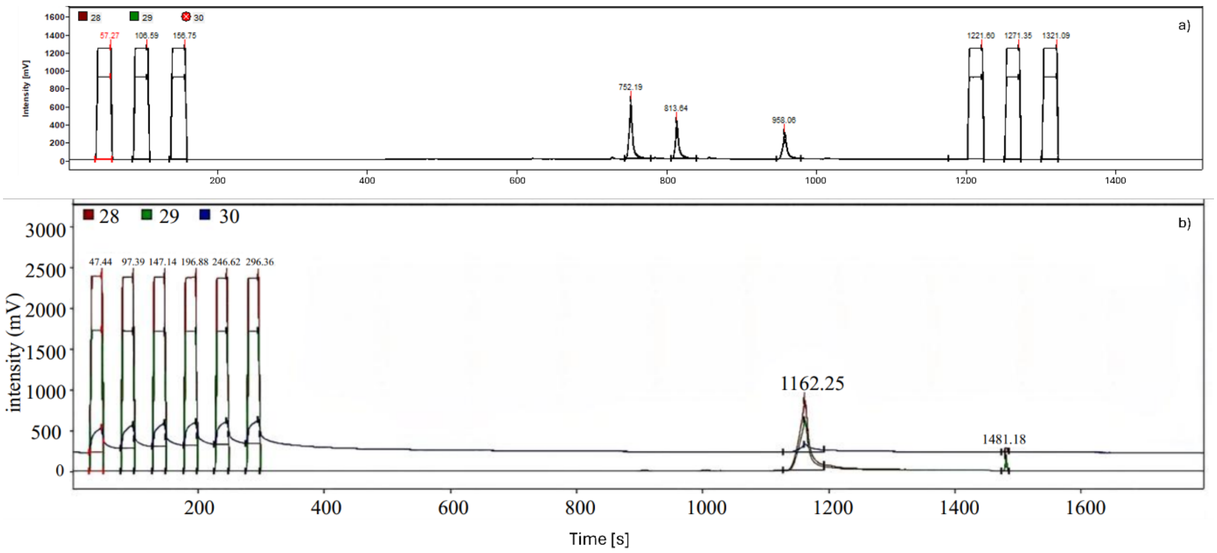

Figure S5: GC-IRMS chromatograms of $\delta^{15}N$ measurements; a) 20 nmol N on column of SMX (t = 752 s), sulfadiazine (t = 813 s), and sulfadimethoxine (t = 958 s) *at TUM*, and b) 66 nmol N on column of SMX (t = 1162 s) *at CAS.*

S7 Derivatization-GC-IRMS for Carbon Isotope Analysis

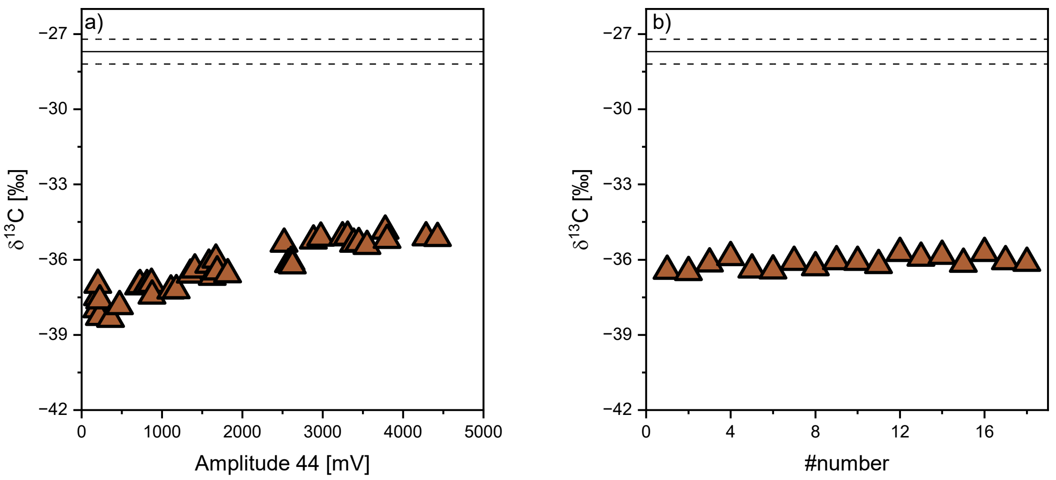

Figure S6: Carbon isotope analysis of derivatized SMX *at CAS*; a) δ^{13}C showing dependence on the amplitudes, b) reproducibility of δ^{13}C values from derivatized SMX measured with GC-IRMS, multiple injections over 3 months; note: in all graphs the black lines show the EA-IRMS value of non-derivatized SMX and its tolerated standard deviation of ±0.5‰ (black dashed lines).

S8 EA-IRMS vs. Derivatization-GC-IRMS of Further Sulfonamides

Table S3: Comparison of δ^{15}N values from EA-IRMS and derivatization-GC-IRMS measurements of sulfadiazine, sulfadimethoxine, sulfadimidine, and sulfathiazole.

Entry	Chemical	Batch number	Laboratory	$\delta^{15}N_{EA\text{-}IRMS}$ [‰]	$\delta^{15}N_{GC\text{-}IRMS}$ [‰]
1	Sulfadiazine	BCCB6548	TUM	−2.4 ± 0.0	−3.5 ± 0.1
2	Sulfadimethoxine	BCCK4645	TUM	−3.6 ± 0.0	−4.1 ± 0.1
3	Sulfadimidine	BCCH7734	TUM	−2.3 ± 0.1	−3.6 ± 0.3
4	Sulfathiazole	BCCG6472	TUM	−3.2 ± 0.2	−1.3 ± 0.2

S9 EA-IRMS vs. Derivatization-GC-IRMS of SMX from Different Suppliers

Table S4: Summary of $\delta^{15}N$ values from EA-IRMS and derivatization-GC-IRMS measurements of sulfamethoxazole from different suppliers.

Entry	Chemical	Lot number	Laboratory	$\delta^{15}N_{EA\text{-}IRMS}$ [‰]	$\delta^{15}N_{GC\text{-}IRMS}$ [‰]
A	Sulfamethoxazole	D0037162	CAS	−2.2 ± 0.1	−3.0 ± 0.1
B	Sulfamethoxazole	G991644	CAS	−2.5 ± 0.0	−3.2 ± 0.1
C	Sulfamethoxazole	V23EN-NI	CAS	−4.5 ± 0.1	−5.4 ± 0.3
D	Sulfamethoxazole	L960V11	CAS	−3.5 ± 0.0	−4.2 ± 0.2
E	Pharmaceutical	-	CAS	-	−7.7 ± 0.4
F	Pharmaceutical	-	CAS	-	−7.3 ± 0.2
G	Pharmaceutical	-	CAS	-	−7.3 ± 0.5
H	Sulfamethoxazole	BCCH3594	TUM	1.0 ± 0.1	1.1 ± 0.1

S10 Recoveries and derivatization-GC-IRMS Analysis from Surface Water

Table S5: Recoveries and $\Delta\delta^{15}N$ value of SMX from extracts of ultrapure and surface water (from Xuanwu Lake in Nanjing) spiked with 10 mg L^{-1} using the SPE procedure.

Entry	Water type	Recovery[a]	$\Delta\delta^{15}N_{GC\text{-}IRMS}$ [‰][b]
1	Ultrapure Water	99 ± 4%	n.a.[c]
2	Surface Water	102 ± 1%	0.3 ± 0.3

[a]determined by HPLC,
[b]deviation of the value of an extract from a measured standard;
[c]n.a. = not analyzed.
